# *HER2* and *BRAF* mutation in colorectal cancer patients: a retrospective study in Eastern China

**DOI:** 10.7717/peerj.8602

**Published:** 2020-02-12

**Authors:** Xiangyan Zhang, Jie Wu, Lili Wang, Han Zhao, Hong Li, Yuhe Duan, Yujun Li, Ping Xu, Wenwen Ran, Xiaoming Xing

**Affiliations:** 1 Department of Pathology, Affiliated Hospital of Qingdao University, Qingdao, China; 2 Department of Pediatrics, Affiliated Hospital of Qingdao University, Qingdao, China; 3 Department of Obstetrics, Laixi People’s Hospital, Qingdao, China

**Keywords:** Human epidermal growth factor receptor 2 gene, *BRAF* mutation, Colorectal cancer, Prognosis

## Abstract

**Objective:**

To investigate the frequency and prognostic role of the human epidermal growth factor receptor 2 gene (*HER2*) and *BRAF* V600E gene mutation in Chinese patients with colorectal cancer (CRC).

**Methods:**

Clinicopathological and survival information from 480 patients with stage I–III CRC were reviewed and recorded. *HER2* amplification was analyzed by immunohistochemistry (IHC) and fluorescence in situ hybridization (FISH), *BRAF* V600E mutation was tested by IHC and Sanger sequencing. The relationship between *HER2* and *BRAF* V600E mutation status and clinicopathological characteristics and outcomes were determined.

**Results:**

The amplification of *HER2* and *BRAF* V600E mutation were identified in 27 of 480 (5.63%) and 19 of 480 (3.96%) CRC patients, respectively. *HER2* amplification significantly correlated with greater bowel wall invasion (*P* = 0.041) and more advanced TNM stage (I vs. II vs. III; 0 vs 5.78% vs. 7.41%, *P* = 0.013). Patients suffering from tumors with poor differentiation had a higher incidence rate of *BRAF* V600E mutation than those with moderate/well differentiation (7.77% vs 2.92%, *P* = 0.04). *HER2* amplification was an independent prognostic factor for worse disease-free survival (DFS) (HR = 2.53, 95% CI: 1.21–5.30, *P* = 0.014).

**Conclusion:**

The prevalence of *HER2* amplification and *BRAF* V600E mutation in stage I–III CRC patients in Chinese was 6% and 4%, respectively, and *HER2* amplification appeared to be associated with a worse DFS. More comprehensive molecular classification and survival analysis are needed to validate our findings.

## Introduction

Colorectal cancer (CRC) is one of the most common malignant tumors in China, with 376,300 new cases and 191,000 disease-related deaths in 2015 (Chen et al., 2016). The outcome of CRC patients has significantly improved over the past decades, but the identification of clinically actionable oncogenic drivers and related predicted biomarkers are largely elusive.

Previous studies have evaluated variety of genetic changes that appear to influence the prognosis of CRC patients, including microsatellite instability (MSI), *RAS* mutation, *BRAF* mutation, the human epidermal growth factor receptor 2 gene (*HER2*) (De Roock et al., 2010; Liu et al., 2017, 2018; Noda et al., 2018; Salvia, Lopez-Gomez & Garcia-Carbonero, 2018). *HER2* gene, which is located on chromosome 17q21, is a tyrosine kinase receptor and encodes for a 185-kDa transmembrane protein (Salvia, Lopez-Gomez & Garcia-Carbonero, 2018). *HER2* gene has been evidenced as a proto-oncogene and identified in many cancer types, including breast, gastric and CRC (Calhoun & Collins, 2015; Sartore-Bianchi et al., 2016; Wakatsuki et al., 2018). *HER2* gene amplification plays a pivotal role in tumor growth and metastasis. In advanced stage CRC, patients with *HER2* amplification tumors were resistant to cetuximab-based treatment (Salvia, Lopez-Gomez & Garcia-Carbonero, 2018). Therefore, the accurate assessment of *HER2* gene amplification status in CRC appears to be particularly important for patients who might undertake this specific targeted therapy.

*BRAF* gene is another important molecular marker for malignancies. *BRAF* gene mutation can activate the RAF/MAPK pathway independently of epidermal growth factor receptor (*EGFR*) activation, leading to poor response to *EGFR* monoclonal antibody (Cetuximab) (De Roock et al., 2010). In additional, detection of *BRAF* mutation is useful to distinguish sporadic MSI CRCs from Lynch syndrome (Loughrey et al., 2007), and the presence of *BRAF* mutation is associated with worse prognosis in metastatic CRC (mCRC) patients (Boursault et al., 2013; Saridaki et al., 2013). But in early stage CRC patients, the prognostic role of *BRAF* mutations is controversial (Gallo et al., 2019; Smeby et al., 2018). A study conducted by European scholars showed *BRAF* mutation was an independent prognostic factor in stage II and III CRC (Fariña-Sarasqueta et al., 2010), and a meta-analysis based on randomized clinical trials showed *BRAF* mutation patients presented poor response to adjuvant chemotherapy (Zhu et al., 2016); however, Chinese scholars demonstrated *BRAF* mutation did not have prognostic value in stage II and III CRC patients (Shen et al., 2016).

Several studies had reported the frequency of *BRAF* mutation in CRC patients, but the number of samples was limited in most of studies. The mutation rate was 5–20% in western countries (Nazemalhosseini-Mojarad et al., 2019; Smeby et al., 2018). But in Chinese, only 5% CRC patients harbored *BRAF* mutation (Shen et al., 2011; Ye et al., 2015; Yunxia et al., 2010). Moreover, the predictive value about this gene in Chinese patients with early stage CRC was also unclear. In addition, information about *HER2* amplification in Chinese CRC patients was limited (Li et al., 2011b). Some studies (Laurentpuig et al., 2016; Stahler et al., 2017) demonstrated patients with *HER2* amplification tumor had a worse survival, but other studies (Pietrantonio et al., 2017; Richman et al., 2016) argued no meaningful relationships between this marker and survival. Therefore, in the present study, we analyzed the *HER2* amplification and *BRAF* V600E mutation status of CRC patients to evaluate possible associations between *HER2* and *BRAF* V600E mutation and the clinicopathological characteristics in primary stage I–III CRC, and we also attempted to explore the prognostic role of *HER2* and *BRAF* V600E mutation.

## Materials and Methods

Four hundred and eighty formalin-fixed, paraffin-embedded tumor specimens from stage I–III CRC patients who underwent primary surgical resection from 2014 to 2016 in the Affiliated Hospital of Qingdao University were selected for this study. Patients who had undergone preoperative radiotherapy, chemotherapy and/or EGFR-targeted therapy were not included in this study. The clinic and pathologic variables were collected as previous description (Zhang et al., 2018a). The patients were followed up until December 2018, and the data concerning cancer recurrence and patient survival were collected. The study was approved by the Ethics Committee of the Affiliated Hospital of Qingdao University (QDFY-20130049).

### *HER2* amplification analysis by immunohistochemistry and fluorescence in situ hybridization

**Figure 1:**
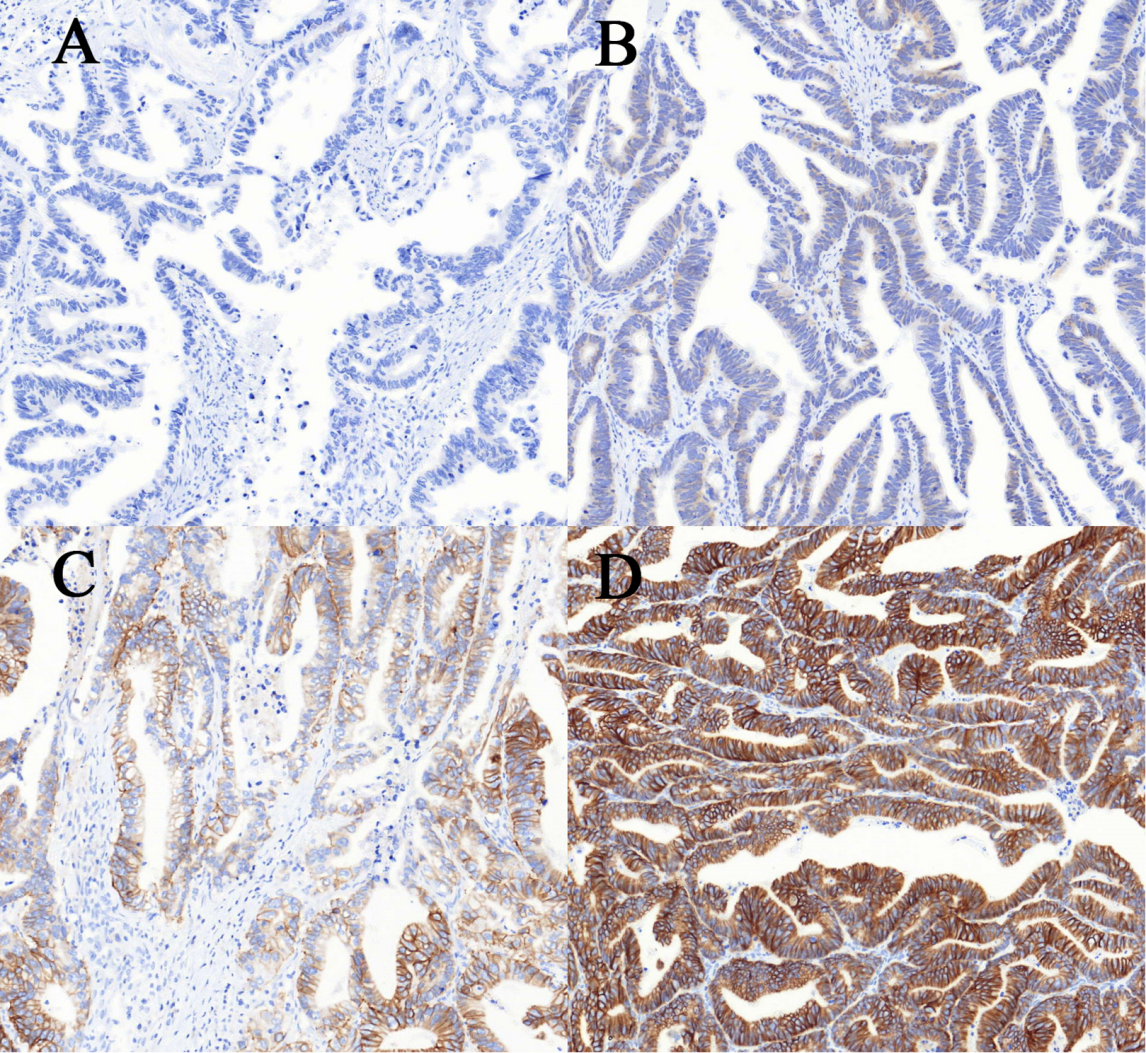
Immunohistochemical staining for human epidermal growth factor receptor 2 (*HER2*) in colorectal cancer. (A–D) The intensity of staining was scored as negative (0, A), weak (1+, B), moderate (2+, C) and strong (3+, D). Allimages are at 100× magnification.

Immunohistochemistry (IHC) staining was performed on an Automated Staining System (BenchMark XT, Ventana Medical Systems, Inc., Oro Valley, AZ, USA). In brief, after deparaffinization and rehydration, paraffin-embedded tissue sections were blocked with CC1 citrate buffer (pH 6.0; Ventana) for 30 min, and incubated with *HER*2 specific monoclonal rabbit antibody (clone 4B5, Ventana Medical Systems Inc., Oro Valley, AZ, USA, working solution) at 37 °C for 32 min. Then, the tissue sections were incubated with 3,3′-diaminobenzidine (DAB) for 4 min. Counterstaining was performed with hematoxylin and bluing reagent for 4 min. The results were analyzed by two pathologists according to the scoring criteria described by Bartley et al. (2017). The IHC staining was scored: 0 (no staining or membrane staining in less than 10% of tumor cells), 1+ (faint/barely visible membrane staining in at least 10% of cells or staining in parts of their membrane), 2+ (weak to moderate complete, basolateral, or lateral membrane staining in at least 10% of tumor cells), 3+ (strong complete or basolateral membrane staining in at least 10% of tumor cells) (Figure 1).

**Figure 2:**
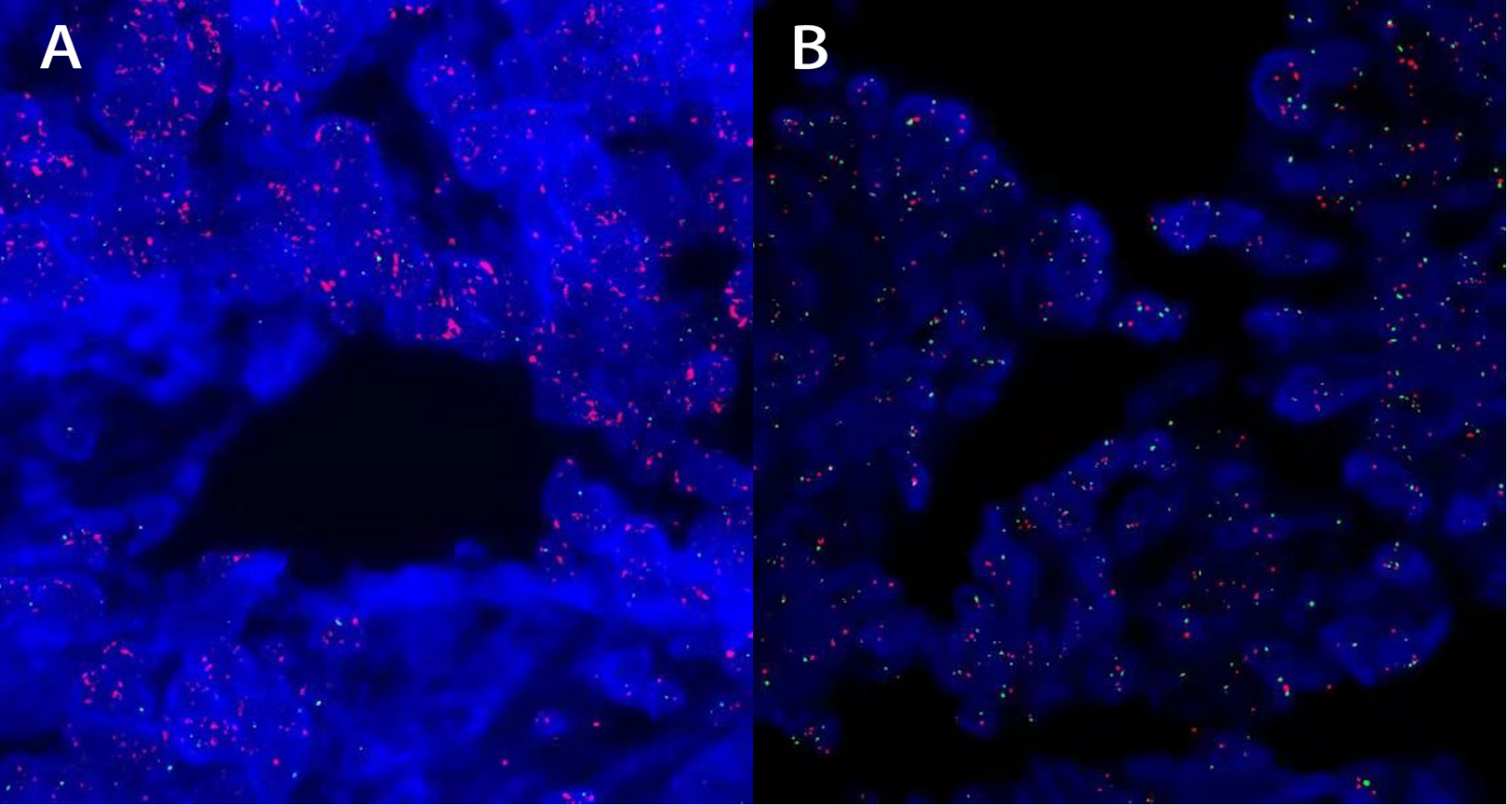
FISH staining for human epidermal growth factor receptor 2 (*HER2*) in colorectal cancer. (A and B) The intensity of staining was scored as positive: *HER2*/CEP17 ratio was 2.3 in 20 tumor nuclei (A) and negative: *HER2*/CEP17 ratio was 1.58 in 20 tumor nuclei (B). All images are at 1,000× magnification.

Samples with 2+ to 3+ IHC staining were retested by fluorescence in situ hybridization (FISH), using the PathVysion *HER2* DNA probe kit and procedure (Vysis/Abbott, Abbott Park, IL, USA). After deparaffinizated and rehydration, paraffin-embedded tissue sections (5 μm thick) were pretreatment at 82 °C for 10 min, then blocked with proteinase at 37 °C for 30 min. After 70% alcohol, tissue sections were denaturation at 75 °C for 10 min and hybridization with *HER2* DNA probe (10 µl) at 37 °C for 24 h, then counterstaining with 4′,6-diamidino-2-phenylindole (DAPI). The scoring for in situ hybridization was performed by counting *HER2* (labeled with Spectrum-Orange) and CEP17 (chromosome 17 enumeration probe labeled with Spectrum-Green) signals from no less than 20 non-overlapping tumor nuclei. Non-tumor tissue (normal colon mucosa) was used as an internal negative control. Samples with a *HER2*/CEP17 ratio ≥2.0, or the presence of tight gene clusters as recently reported (Valtorta et al., 2015) were considered as amplified, otherwise samples were defined as negative for *HER2*. FISH staining was evaluated by two different pathologists to ensure consistency (Figure 2).

### Analysis of *BRAF* V600E mutation by immunohistochemistry and Sanger sequencing

**Figure 3:**
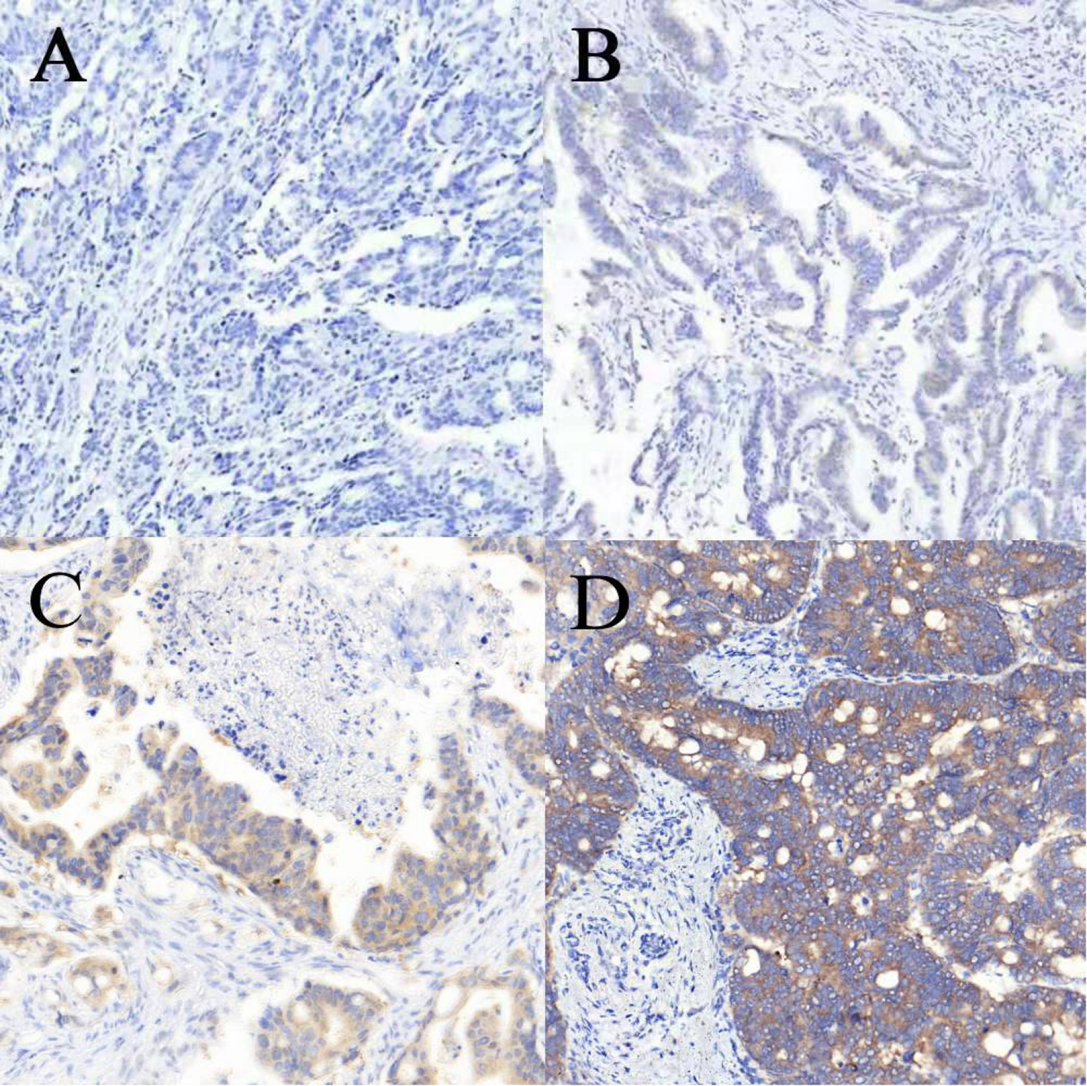
Immunohistochemical staining for *BRAF* V600E in colorectal cancer. (A–D) The intensity of staining was scored as negative (0, A), weak (1+, B), moderate (2+, C) and strong (3+, D). All images are at 100× magnification.

Immunohistochemistry staining and Sanger sequencing for *BRAF* mutation were performed as previously described by Zhang et al. (2018b). In brief, after deparaffinization and rehydration, paraffin-embedded tissue sections (3 μm thick) were blocked with 3% hydrogen peroxide for 4 min at room temperature, treated with heat-induced antigen retrieval CC1 solution for 32 min, and incubated with *BRAF* V600E specific monoclonal mouse antibody (clone VE1, item code 790–4855, Ventana Medical Systems Inc., Oro Valley, AZ, USA, working solution) at 37 °C for 32 min. Then, the tissue sections were incubated with OptiView HRP Linker for 12 min, OptiView HRP multimer for 12 min, and developed with DAB for 4 min. Counterstaining was performed with hematoxylin and bluing reagent for 4 min. The results were analyzed by two pathologists according to the scoring criteria described by Zhang et al. (2018b). *BRAF* V600E expression within the cytoplasm was subjectively graded as: 0, no cytoplasmic staining visualized at any magnification; 1+, weak, requiring a 10× or greater objective to recognize flavescent staining on the section; 2+, moderate, easy to recognize yellow staining with a 10× objective; and 3+, strong brown staining with a 10× microscopic objective (Figure 3).

**Figure 4:**
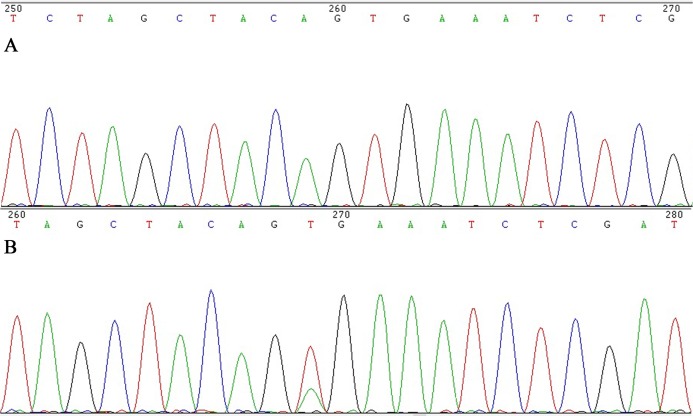
Sanger sequencing for *BRAF* V600E in colorectal cancer. (A), *BRAF* V600E wildtype by Sanger sequencing, (B) *BRAF* V600E *mutation* by Sanger sequencing.

All CRC cases with cytoplasmic staining were retested by Sanger sequencing analysis to exclude false positive. DNA was extracted using the Blood and Tissue DNA retraction Kit (Tiangen Inc., Beijing, China). Primers were designed to amplify the V600E point mutation. The sequence of the forward primer was 5′-TCATCCTAACACATTTCAAGCC-3′ and the reverse primer was 5′-GTAAAACGACGGCCAGTTTTGTGAATACTGGGAACTATGAAA-3′. PCR amplification conditions: 95 °C for 3 min, followed by 45 cycles of 94 °C for 15 s, 60 °C for 45 s, with a final cooling time for 1 min at 25 °C. The PCR products were purified with QIAquick Gel Extraction Kit (Qiagen). The cycling conditions were as follows: 96 °C for 1 min, followed by 30 cycles of 96 °C for 10 s, 50 °C for 5 s, with a final extension at 60 °C for 2 min. Purified products were then run on an ABI 3500 DX Genetic Analyzer (Applied Biosystems, Foster City, CA, USA) and analyzed using software supplied by the manufacturer (Zhang et al., 2018b). Both forward and reverse strands were sequenced. The sequences were compared with the database sequence in GenBank sequence database (HGNC: 1097) (Figure 4).

### Statistical analysis

Results were analyzed with SPSS 19.0 (SPSS, Inc., Chicago, IL, USA). For comparison of the frequencies among groups, Chi-square test and Fisher exact test were used. Survival curves for disease-free survival (DFS) and overall survival (OS) were estimated using Kaplan-Meier analysis with the log-rank test. Multivariable analysis was performed using Cox regression. Probability (*P*) value <0.05 was considered as statistical significance.

## Results

### *HER2* status and associations with clinicopathological characteristics

HER2 IHC scores of 3+, 2+, 1+ and 0 were observed in 10 (2.1%), 54 (11.3%), 86 (17.9%) and 330 (68.7%) tumors, respectively. *HER2* gene amplification was seen in 27 samples (9 cases with *HER2* IHC scores of 3+, 18 cases with *HER2* IHC scores of 2+) (Supplemental Files). The amplification rate of *HER2* in CRC was 5.63% (27/480). *HER2* status and clinicopathological characteristics, including age, gender, tumor location, tumor size, histological characteristics, TNM stage and family medication history are shown in Table 1. *HER2* amplification was significantly correlated with greater bowel wall invasion (*P* = 0.041) and more advanced TNM stage (I vs II vs III; 0 vs 5.78% vs 7.41%, *P* = 0.013). Although *HER2* amplification tumors were present more often in patients with CRC family history, there is no significant statistical difference in this study (10.34% vs 6.59%, *P* > 0.05).

**Table 1:** Correlations between *HER2* and *BRAF* gene mutation and clinicopathological characteristics (*n* = 480).

Characteristics	Number	*BRAF*		*P*	*HER2*		*P*
		Mutation	%		Amplification	%	
Gender							
Male	288	10	3.47	0.51	18	6.25	0.55
Female	192	9	4.69		9	4.69	
Age (year)							
≤50	63	3	4.76	0.72*	5	7.94	0.38*
>50	417	16	3.84		22	5.28	
Location							
Right side colon	105	3	2.86	0.19*	5	4.76	0.99
Left side colon	87	7	8.05		6	6.90	
Rectum	269	9	3.35		16	5.95	
Mucin production							
With	415	15	3.61	0.31*	23	5.54	0.77*
Without	65	4	6.15		4	6.15	
Tumor differentiation							
Poor	103	8	7.77	0.04*	5	4.85	0.71
Moderate/well	377	11	2.92		22	5.84	
Tumor stage							
I	66	2	3.03	0.62	0	0.00	0.013*
II	225	11	4.89		13	5.78	
III	189	6	3.17		14	7.41	
Bowel wall invasion (T)							
T1+T2	90	2	2.22	0.55*	1	1.11	0.041*
T3+T4	390	17	4.36		26	667	
Tumor diameter							
<5 cm	261	7	2.68	0.18	14	5.36	0.97
≥5 cm	219	12	5.48		13	5.94	
Lymph node metastasis (N)							
With	189	6	4.47	0.48	13	6.88	0.17
Without	291	13	3.17		14	4.81	
Lymphovascular invasion							
No	332	14	4.22	0.66	17	5.12	0.47
Yes	148	5	3.38		10	6.76	
Alcohol intake							
Ever	99	2	2.02	0.39*	4	4.04	0.44
Never	381	17	4.46		23	6.03	
Smoking							
Ever	129	3	2.33	0.27	8	6.20	0.74
Never	351	16	4.56		19	4.69	
Cancer family history							
Yes	92	3	3.26	0.99	7	7.61	0.61
No	119	4	3.36		8	6.72	
Unknown	269						
Colorectal family history							
Yes	29	0	0.00	0.59*	3	10.34	0.49*
No	182	7	3.85		12	6.59	
Unknown	269						
MSI status							
MSI	72	4	5.56	0.51*	4	5.56	0.98*
MSS	408	15	3.68		23	5.63	
*RAS* status							
Mutation	207	0	0	0.001	2	3.48	0.001
Wildtype	273	19	6.95		25	9.12	

**Note**:

* Fisher exact test were used.

### *BRAF* V600E status and associations with clinicopathological characteristics

Immunohistochemistry testing showed 26 cases with positive staining and 454 cases with negative staining. The distribution of positive IHC staining was 3+ in 12 cases, 2+ in 8 cases, and 1+ in 6 cases in CRCs (Supplemental Files). Sanger sequencing showed 19 samples (12 cases with 3+ and 7 cases with 2+) harbored *BRAF* V600E mutation, so the mutation rates of *BRAF* V600E were 3.96% (19/480). Patients suffering from tumors with poor differentiation had a higher incidence rate of *BRAF* V600E mutation compared with those having tumors with moderate/well differentiation (7.77% vs 2.92%, *P* = 0.04). No significant difference between *BRAF* V600E mutation and other clinicopathological characteristics was found in present study (Table 1).

### Prognostic value of *HER2* amplification and *BRAF* V600E mutation in stage I–III CRC

Univariable analysis by Kaplan-Meier survival analysis and log-rank test was performed to evaluate the significance of clinicopathological factors for DFS and OS. We found that factors with statistical significance for DFS were age (*P* = 0.04), tumor differentiation (*P* = 0.002), bowel wall invasion (*P* = 0.04), lymph node metastasis (*P* = 0.03), lymphovascular invasion (*P* = 0.001) and *HER2* amplification (*P* = 0.007). Factors that were statistically significant for OS were age (*P* = 0.04), tumor differentiation (*P* = 0.03), tumor stage (*P* = 0.001), bowel wall invasion (*P* = 0.01), lymph node metastasis (*P* = 0.001) and lymphovascular invasion (*P* = 0.001) (Table 2).

**Table 2:** Univariate analysis of prognostic factors influencing disease free survival (DFS) and overall survival (OS) in stage I–III colorectal cancer.

Characteristics	DFS	*P*	OS	*P*
	HR (95% CI)		HR (95% CI)	
Gender				
Male vs female	1.13 [0.71–1.83]	0.6	1.18 [0.7–2.0]	0.54
Age (year)				
≤50 vs >50	0.49 [0.25–0.97]	0.04	0.47 [0.22–0.97]	0.04
Location				
Right side colon vs left side colon vs rectum		0.73		0.85
Mucin production				
With vs without	1.75 [0.89–3.47]	0.11	1.8 [0.85–3.80]	0.12
Tumor differentiation				
Poor vs moderate/well	2.56 [1.40–4.68]	0.002	2.08 [1.09–3.99]	0.03
Tumor stage				
I vs II vs III		0.06		0.001
Bowel wall invasion (T)				
T1+T2 vs T3+T4	0.54 [0.30–0.97]	0.04	0.44 [0.23–0.85]	0.01
Tumor diameter				
≤5 cm vs >5 cm	1.17 [0.73–1.88]	0.5	1.11 [0.66–1.86]	0.7
Lymph node metastasis (N)				
Without vs with	0.59 [0.36–0.95]	0.03	0.39 [0.23–0.66]	0.001
Lymphovascular invasion				
No vs yes	0.41 [0.24–0.69]	0.001	0.42 [0.23–0.75]	0.001
Alcohol intake				
Ever vs never	1.1 [0.62–1.94]	0.75	0.97 [0.52–1.81]	0.91
Smoking				
Ever vs never	1.3 [0.77–2.19]	0.32	0.99 [0.55–1.77]	0.97
Cancer family history				
Yes vs no	1.86 [0.92–3.76]	0.09	1.91 [0.86–4.28]	0.11
Colorectal family history				
Yes vs no	1.12 [0.41–3.06]	0.83	0.75 [0.23–2.44]	0.64
MSI status				
MSI vs MSS	1.14 [0.58–2.25]	0.69	0.98 [0.46–2.06]	0.95
*RAS* status				
*RAS* mutation vs *RAS* wildtype	1.35 [0.84–2.19]	0.21	1.45 [0.86–2.25]	0.16
*BRAF* status				
*BRAF* mutation vs *BRAF* wildtype	3.41 [0.91–12.74]	0.07	1.08 [0.25–4.68]	0.92
*HER2* status				
*HER2* amplification vs *HER2* negative	3.97 [1.35–11.72]	0.007	2.98 [0.92–9.67]	0.08

**Figure 5:**
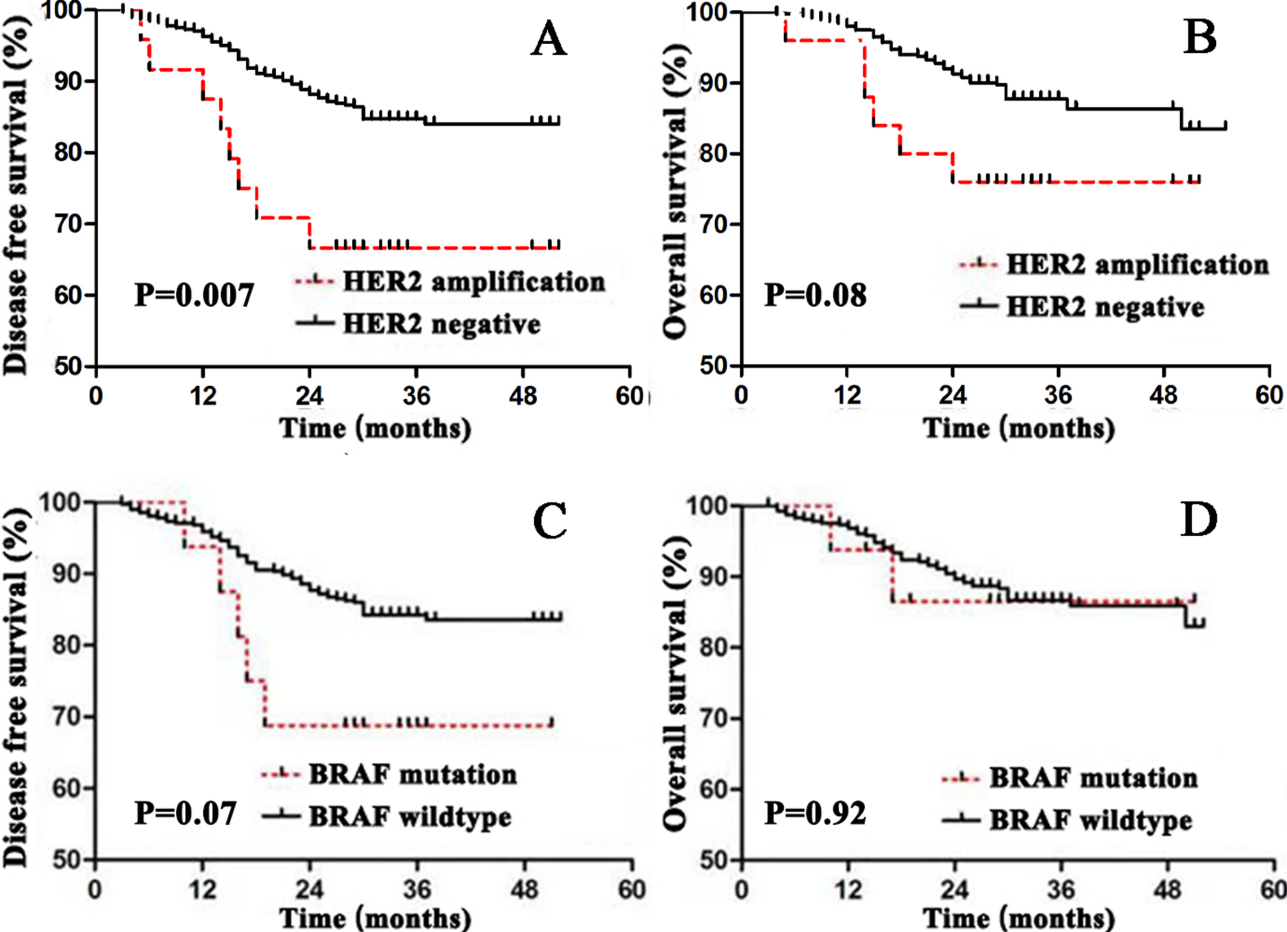
Survival curves for disease free survival (DFS) and overall survival (OS) in stage I–III colorectal cancer according to *HER2* or *BRAF* status. (A) DFS according to *HER2* status; (B) OS according to *HER2* status; (C) DFS according to *BRAF* status; (D) OS according to *BRAF* status.

Patients with *HER2* amplification tumors were found to have significantly worse DFS (*P* = 0.007) (Figure 5A). To determine the prognostic value independent of age distribution, tumor differentiation, bowel wall invasion, lymph node metastasis, lymphovascular invasion and *HER2* amplification were entered into a Cox regression model. Multivariate Cox regression analysis showed *HER2* amplification was an independent risk factor for worse DFS, tumors with *HER2* amplification were associated with a 2.53-fold increase in risk of cancer recurrence (HR = 2.53, 95% CI [1.21–5.30], *P* = 0.014) (Table 3).

**Table 3:** Independent prognostic factors correlating with disease free survival (DFS) in stage I–III colorectal cancer by Cox’s regression analysis.

Characteristics	HR (95% CI)	*P*
*HER2* amplification	2.53 [1.21–5.30]	0.014
Tumor poor differentiation	1.91 [1.16–3.14]	0.011
Lymphovascular invasion	2.15 [1.34–3.44]	0.001

## Discussion

HER2 and *BRAF* mutation are important for clinical treatment and prognosis evaluation in cancer patients. As we know, *HER2* has been found to be a predictive marker to *HER2*-targeted therapy in breast and gastric cancer; therefore, routine test for *HER2* status is mandatory in these tumors (Calhoun & Collins, 2015; Jiang et al., 2018). The frequency of *BRAF* mutation in CRCs ranges from 5% to 15%, and *BRAF* mutation has been demonstrated to be a worse prognostic factor in mCRC (Boursault et al., 2013) and should be tested for mCRC patients before MoAb treatment (De Roock et al., 2010). No evidence has convinced that *HER2* gene amplification is a prognostic factor for CRC. Laurentpuig et al. (2016) and Stahler et al. (2017) demonstrated patients with *HER2* amplification tumor had a worse survival, but some recent studies (Pietrantonio et al., 2017; Richman et al., 2016) argued no meaningful relationships between this marker and survival. Several studies had reported the frequency of *BRAF* mutation in Chinese CRC patients, but the number of samples was limited in most of the studies (Li et al., 2011a; Mao et al., 2012). Thus, we designed this study in Chinese population to explore the relationship between *HER2* amplification and *BRAF* mutation and clinicopathological parameters, and to evaluate prognostic and predictive values of *HER2* amplification and *BRAF* mutation for CRC.

In our study, the *HER2* gene amplification rate is 5.63%, similar to some other studies (Hyman et al., 2018; Jeong et al., 2016; Laurentpuig et al., 2016; Richman et al., 2016). However, report from Korea (Dong et al., 2007) showed the protein expression rate was 47%. There are several possible reasons for this discrepancy such as ethnic diversity and test methods, but the most likely reason for the divergent findings might be the different scoring systems. Dong et al. (2007) judged only cytoplasmic staining in >20% of tumor cells to be positive, so there was relatively higher positive rate in CRC. Nowadays, there are two different scoring systems for *HER2* IHC in CRC, gastroesophageal adenocarcinoma criteria (GEA criteria) and *HER2* Amplification for Colorectal Cancer Enhanced Stratification diagnostic criteria (HERACLES criteria) (Valtorta et al., 2015). The different criteria of membrane positivity may also cause for the conflicting results, Liu et al. (2019) argued *HER2* status evaluated by the HERACLES criteria showed survival predictive for stage II–III CRC, but the results were not represented based on the GEA diagnostic criteria. In present study, the GEA criteria was used, and 33.3% (18/54) samples with IHC staining 2+ were confirmed to harbor *HER2* gene amplification, but Wang et al. (2019) judged only 20% of tumors with *HER2* IHC 2+ staining showing gene amplification based on this criteria. IHC is a semiquantitative method and may be influenced by subjective perception of pathologists frequently, so exactly *HER2* positive standardized by IHC in CRC is urgently needed.

In our current study, we found *HER2* gene amplification was related to bowel wall invasion and advanced tumor stage, but several other studies have failed to show such relationship. Li et al. (2011b) reported an association between *HER2* expression and tumor size and distant metastases, and a recent meta-analysis showed *HER2* amplification was associated with lymph node metastasis and advanced tumors stage (Sun et al., 2016). The number of studies suggests that *HER2* may play some role in tumor progression and would be a valuable prognostic factor for CRC patients (Laurentpuig et al., 2016; Stahler et al., 2017). But in other studies, *HER2* gene amplification was higher in patients with more advanced stage or distant metastases, and no significant difference in prognosis for CRC patients (Pietrantonio et al., 2017; Wang et al., 2019).

The prognostic value of *HER2* amplification in CRC patients has been widely investigated, but no rationale had been obtained. A study from Germany indicated that *HER2* amplification resulted in poorer prognosis in all stage CRC (Ingold Heppner et al., 2014), and Laurentpuig et al. (2016) found that *HER2* amplification was significantly associated with worse prognosis in patients with stage III CRC. Moreover, some studies showed patients with *HER2* wild-type tumors have positive OS compared with those tumors containing amplified *HER2* due to benefit from anti-EGFR monoclonal antibody, and the targeted management may be an influential factor in patients with advanced CRC (Pietrantonio et al., 2017; Salvia, Lopez-Gomez & Garcia-Carbonero, 2018). In this study, we demonstrated that *HER2* amplification was independently associated with worse survival in DFS, considering that none of our patients received anti-EGFR treatment before recurrence, the influence in survival due to targeted management could be excluded. Therefore, we confirmed *HER2* amplification tumors had a higher propensity to recurrence and metastasize, and *HER2* amplification was an independent prognostic factor for DFS in stage I–III CRC.

In the present study, the mutation rates of *BRAF* was 3.96%, the *BRAF* mutation rate is similar to that previously reported in Iranian (5.8%, 15/258) (Nazemalhosseini-Mojarad et al., 2019), but significantly lower than the value of 16% among 1185 CRC patients from Norway (Smeby et al., 2018). On the other hand, the association between *BRAF* mutations and clinicopathological characteristics has not been well known in Chinese CRC patients due to the insufficient number of CRCs with *BRAF* mutation. Nonetheless, reports from other countries have demonstrated that tumors with *BRAF* mutation are associated with tumor location, tumor grade, and mucinous production (Nazemalhosseini-Mojarad et al., 2019; Zlobec et al., 2010), our data confirmed *BRAF* mutations was only association with tumor differentiation. Several factors may be related to these differences, such as sample size, the distribution of age, stage, as well as racial and/or environmental differences.

Most previous studies demonstrated *BRAF* mutation was related to poorer outcomes (Kadowaki et al., 2015; Smeby et al., 2018; Toon et al., 2014). However, this evidence is mainly based on studies in western country, little is known about the prognostic role of *BRAF* mutations in eastern Chinese populations. Contrary to some previous studies, no associations of *BRAF* mutation with DFS and OS were found in eastern Chinese CRC patients in our study. In additional, in patients with mCRC, *BRAF* mutation acted as worse prognostic markers, and patients with *BRAF* mutations were less likely to achieve response to treatment with panitumumab or cetuximab (Di Nicolantonio et al., 2008; Saridaki et al., 2013; Benvenuti et al., 2007). We did not evaluate the effect of panitumumab or cetuximab treatment after tumors achieved distant metastases, so the predictive value of *BRAF* mutation for monoclonal antibody treatment in stage IV CRC patients was unexplored. This is the preliminary study to explore the prognostic value of *BRAF* mutation in this area and further studies based on larger sample size and longer follow-up time are needed to confirm this finding.

## Conclusion

In summary, we investigated *HER2* and *BRAF* gene status in a series of stage I–III CRC patients in eastern China. Our data show the prevalence of *HER2* and *BRAF* mutation was 5.63% and 3.96%, respectively. *HER2* amplification appears to be associated with a worse DFS. More comprehensive molecular classification and survival analysis are needed to validate our findings.

## Supplemental Information

10.7717/peerj.8602/supp-1 Supplemental Information 1 Data of 480 CRC patients.

## Additional Information and Declarations

### Data Availability

The following information was supplied regarding data availability: The raw data is available in the Supplemental File.
